# Response to “Comment on the systematic review of TROPIS for anal fistula”

**DOI:** 10.1016/j.sopen.2025.10.004

**Published:** 2025-10-14

**Authors:** Yang-Tao Chen, Rong Shi, Jing Wang

**Affiliations:** a Department of Anorectal Surgery, The Affiliated People's Hospital of Fujian University of Traditional Chinese Medicine, Fuzhou, 350004, China

Dear Editor,

We appreciate the interest and constructive comments from Dr. Yagnik and colleagues [2] regarding our recent systematic review and meta-analysis on the Transanal Opening of Intersphincteric Space (TROPIS) procedure for anal fistula [1]. Their thoughtful observations allow us to clarify several key points and acknowledge certain limitations in our work.

First, we agree that the predominance of studies from China limits the generalizability of our conclusions. This reflects the current distribution of published evidence rather than a selective inclusion bias, as our search covered multiple international databases without language restrictions. Nonetheless, a broader global dataset would certainly improve representativeness, and we encourage future multicenter collaborations to achieve this.

Second, we acknowledge that many included studies were observational with variable methodological quality. The ROBINS-I and GRADE results clearly indicated this, and we did not intend to overstate the certainty of our findings. Our statement that TROPIS “may become a new reference standard” was meant to highlight its emerging potential rather than to assert established superiority. We recognize that this phrasing could have been more conservative.

Third, we concur that objective continence assessments such as anorectal manometry or the Garg Incontinence Score would strengthen the evaluation of postoperative function. Unfortunately, these data were seldom available in the published literature, which restricted our analysis. Nevertheless, several studies included in our review reported improved continence outcomes using validated subjective scales, suggesting a favorable trend that warrants further verification.

Finally, we emphasize that the goal of our study was to synthesize available evidence and identify gaps for future research, not to provide definitive clinical recommendations. Constructive academic exchange, such as the letter from Dr. Yagnik and colleagues, contributes meaningfully to refining both methodology and interpretation in this area.

We thank the authors once again for their engagement and critical insights. Continued collaboration and high-quality randomized studies will be essential to determine the precise role of TROPIS in complex anal fistula management.

## CRediT authorship contribution statement

**Yang-Tao Chen:** Conceptualization, Writing – original draft, Writing – review & editing. **Rong Shi:** Supervision, Validation. **Jing Wang:** Supervision, Writing – review & editing.

## Funding

Clinical Research Center for Traditional Chinese Medicine on Anorectal and Perianal Wound Repair of Fujian Province (No. Fujian Science and Technology Department Document 2022Y2011).

## Declaration of competing interest

The authors of this manuscript declare that they have no financial conflicts of interest that could influence the objectivity or integrity of the research presented in this paper. Furthermore, the authors declare that they have no personal or professional relationships that could be perceived as potential conflicts of interest.
